# Periorbital discoid lupus: a rare localization in a patient with systemic lupus erythematosus*

**DOI:** 10.1590/abd1806-4841.20164708

**Published:** 2016

**Authors:** Ozgur Cakici, Remzi Karadag, Huseyin Bayramlar, Seyma Ozkanli, Tugba Kevser Uzuncakmak, Ayse Serap Karadag

**Affiliations:** 1 Istanbul Medeniyet University Goztepe Research and Training Hospital – Istanbul, Turkey; 2 Istanbul Medeniyet University School of Medicine – Istanbul, Turkey

**Keywords:** Eye, Eyelids, Lupus erythematosus, discoid

## Abstract

A 40-year-old female patient with a 5-year history of systemic lupus erythematosus was referred to our policlinic with complaints of erythema, atrophy, and telangiectasia on the upper eyelids for 8 months. No associated mucocutaneous lesion was present. Biopsy taken by our ophthalmology department revealed discoid lupus erythematosus. Topical tacrolimus was augmented to the systemic therapeutic regimen of the patient, which consisted of continuous antimalarial treatment and intermittent corticosteroid drugs. We observed no remission in spite of the 6-month supervised therapy. Periorbital discoid lupus erythematosus is very unusual and should be considered in the differential diagnosis of erythematous lesions of the periorbital area..

## INTRODUCTION

Discoid lupus erythematosus (DLE) is the most common chronic form of lupus erythematosus (LE). DLE is more common than the systemic form of the lupus erythematosus. Clinical features include lesions with squamous and erythematous scaly plaques that may result in atrophic scars, alopecia, or permanent pigmentary changes. The most commonly involved areas are those exposed to sun, such as the face, the "V" region of the neck, and the extensor sides of the arms; periorbital localization is rare.^1^ Isolated eyelid involvement is rarely reported and its diagnosis is difficult due to the typical lack of morphological findings. Delayed diagnosis usually reflects in treatment delays.^2,3^

We report a case of DLE on the eyelid in a patient who had been followed for treatment-resistant systemic LE.

## CASE REPORT

A 40-year-old woman had been followed with the diagnosis of systemic lupus erythematosus (SLE) for 5 years in our rheumatology clinic. She presented photosensitivity, synovitis, arthritis, hemolytic anemia, leucopenia, positive antinuclear antibodies, and positive anti-dsDNA antibodies. According to The American College of Rheumatology (ACR) and the SLICC (Systemic Lupus Collaborating Clinics) criteria, the patient was diagnosed with SLE. She presented to our dermatology clinic with the complaints and signs of erythema, telangiectasia, and mild atrophy of her upper eyelids and left lower eyelid. The complaints about the eyelids had started about 8 months before the patient sought treatment and worsened with sun exposure. Ophthalmologists suspected discoid lupus erythematosus.

**Figure 1:**
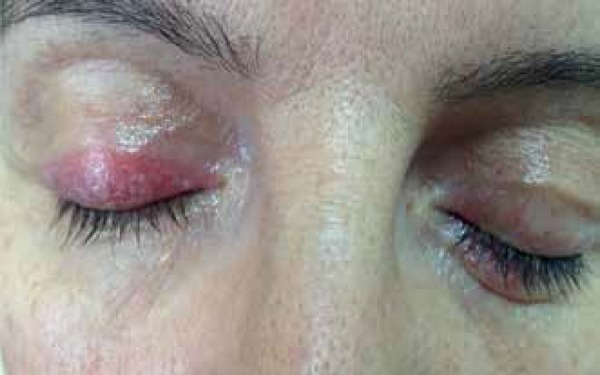
Squamous, erythematous, mildly edematous, and atrophic areas in some parts, and patchy- eyelid lesions

Ophthalmic examination revealed squamous, erythematous, mildly edematous, and atrophic areas. Scaly patches and telangiectasias were seen on both eyelids (5-6 mm x 1-2 mm on the right upper side and 2 mm x 1 mm on the left upper and lower eyelids) (Figure 1). The lesion on the right side covered more than 2/3 of the upper eyelid starting on the nasal side, while the lesion on the left side spread to less than 2/3 of the upper eyelid area, localized on the upper nasal and lower temporal sides. We noted no signs of conjunctivitis, meibomitis, or blepharitis.

**Figure 2:**
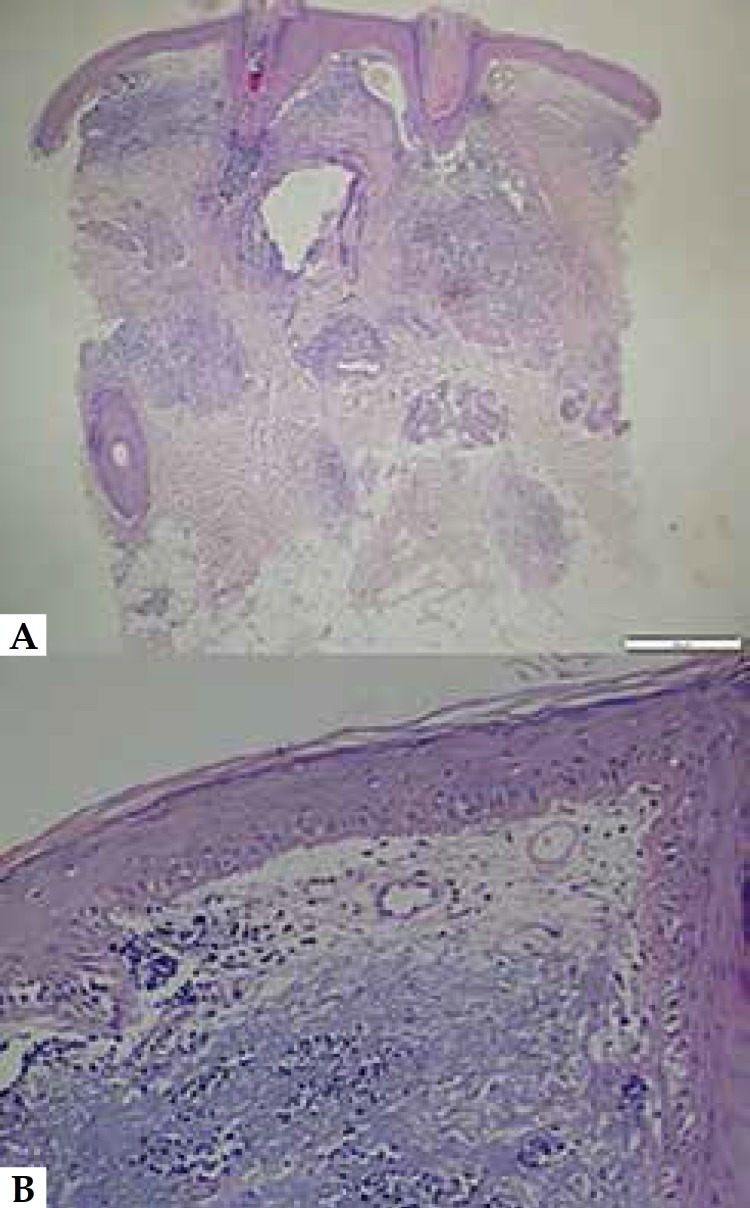
Histological examination revealed epidermal atrophy, vacuolar degeneration, and lymphocytic infiltrations in deep and superficial perivascular and periadnexial areas (A. Hematoxylin - eosin, x4, B. Hematoxylin - eosin, x20)

Routine tests showed normal results. Laboratory investigation yielded positive results for anti-nuclear antibodies and anti-dsDNA tests. We took an excisional biopsy from the right upper eyelid based on a suspected DLE diagnosis. Histological examination revealed epidermal atrophy, vacuolar degeneration, and lymphocytic infiltrations in deep and superficial perivascular and periadnexial areas, confirming the DLE diagnosis (Figure 2). The patient had already been taking hydroxychloroquine 200 mg (orally, twice a day) as a systemic corticosteroid treatment. We prescribed topical corticosteroid for the eyelids for 1 month. Since no improvement was observed with this regimen, we augmented tacrolimus ointment 0.03%. We also advised the patient to avoid sun exposure and to wear sunglasses. We saw no signs of symptom regression during the 6-month follow-up. The patient currently remains under our supervision.

## DISCUSSION

Discoid lupus erythematosus (DLE) is the most common form of chronic cutaneous lupus erythematosus (CCLE) and is mostly seen in women 20-40 years of age. Eyelid involvement is seen in 5%-6% of DLE patients. ^3,5-7^ The disease typically manifests with bilateral eyelid involvement and mucocutaneous lesions.^2^ Cases involving the conjunctiva and the cornea have also been reported. DLE patients presenting with unilateral eyelid involvement are very unusual and its diagnosis is a challenge for physicians since it lacks the typical morphologic signs of CCLE.^2,4,5^ DLE patients may complain of mild pruritus or sometimes pain, but most cases are asymptomatic. Serological and hematological signs are seen in patients with typical involvement.

Delay in diagnosis can lead to eye or eyelid complications – such as periorbital edema, epiphora, trichiasis, conjunctivitis, stromal keratitis, madarosis, ectropion, and entropion. Such complications, in addition to the development of a permanent scar, can disturb the psychological status of the patient.^8^ Some patients presenting with these symptoms received treatment for chronic blepharitis or eczema for many years, as illustrated by Aubaret *et al.* and Duke-Elder *et al.*, who reported cases of DLE presented as chronic blepharitis.^9,10^ In our case, the diagnosis was made 6 months after the beginning of the treatment.

Several diseases may be initially considered in the differential diagnosis of DLE: basal cell carcinoma, squamous cell carcinoma, Bowen's disease, actinic keratosis, contact dermatitis, atopic dermatitis, seborrheic dermatitis, psoriasis, and sarcoidosis.^1,5,8,10^

Treatment of DLE lesions on the eyelid usually involves oral hydroxychloroquine, topical corticosteroid, and avoidance of sun exposure. Some researchers also suggest the use of topical tacrolimus or intralesionary steroid. ^4^ In our case, we observed no healing with the use of systemic antimalarial, topical corticosteroid, or topical tacrolimus medications.

Although DLE on the eyelids typically has a benign course, diagnosis is frequently difficult, resulting in patients receiving treatments for different diagnoses. As a consequence, treatment results may be negative.
